# Tenofovir-Based Highly Active Antiretroviral Therapy Is Associated with Superior CD4 T Cells Repopulation Compared to Zidovudine-Based HAART in HIV 1 Infected Adults

**DOI:** 10.1155/2018/3702740

**Published:** 2018-04-18

**Authors:** Vitus Sambo Badii, Kwame Ohene Buabeng, Thomas Agyarko Poku, Arnold Donkor Forkuo, Bright Boafo Boamah, Stephen Mensah Arhin, Daniel Edem Kpewou

**Affiliations:** ^1^Department of Pharmacology, Faculty of Pharmacy and Pharmaceutical Sciences, Kwame Nkrumah University of Sciences and Technology, Kumasi, Ghana; ^2^Department of Pharmacy Practice, Faculty of Pharmacy and Pharmaceutical Sciences, Kwame Nkrumah University of Sciences and Technology, Kumasi, Ghana; ^3^Suntreso Government Hospital, Kumasi, Ghana; ^4^Department of Biochemistry and Biotechnology, College of Science, Kwame Nkrumah University of Sciences and Technology, Kumasi, Ghana

## Abstract

Tenofovir-based highly active antiretroviral therapy (HAART) is one of the preferred first-line therapies in the management of HIV 1 infection. Ghana has since 2014 adopted this recommendation; however there is paucity of scientific data that reflects the safety and efficacy of the tenofovir-based therapy compared to zidovudine in the Ghanaian health system. This study sought to assess the comparative immune reconstitution potential between tenofovir and zidovudine-based HAART regimens, which includes lamivudine and efavirenz in combination therapy. It also aimed to investigate the adverse drug reactions/events (ADREs) associated with pharmacotherapy with these agents in a total of 106 HAART naïve HIV patients. The study included 80 patients in the tenofovir cohort while 26 patients were on the zidovudine regimen. The occurrence of HIV comorbidities profile was assessed at diagnosis and throughout the study period. The baseline CD4 T cells count of the participants was also assessed at diagnosis and repeated at a median period of five months (range 4–6 months), after commencing treatment with either tenofovir- or zidovudine-based HAART. After five months of the HAART, the tenofovir cohort recorded higher CD4 T cell count change from baseline compared to the zidovudine cohort (*p* < 0.0001). The patients on the tenofovir-based HAART and female sex however appeared to be associated with more multiple ADREs.

## 1. Introduction

The main physiological derangement in HIV infection is an uncontrolled CD4 T cells decline [1–3]. Studies suggest CD4 T cell death via cytolysis, chronic immune activation [3–5], cytotoxic T cells response, and gp120-envelop induced immune reactions [6–8] to partly account for the overall decline. Furthermore, the failure of thymus T cell hemostatic function due to HIV-induced thymopathy and infection of hematopoietic progenitor cells helps to establish this progressive CD4 T cell decline in chronic HIV infection [3, 6]. As a result, people living with HIV/AIDS (PLWHA) often present with low CD4 T cell count which permits dormant human flora and other potential pathogens to revert to overt forms causing opportunistic infections and rare cancers such as Kaposi sarcoma [3, 9, 10].

The advent of Highly Active Antiretroviral therapy (HAART) has revolutionized the management of PLWHA over the past decades [10, 11]. It inhibits viral replication and suppresses HIV RNA with resultant regression in HIV associated immune activation and CD4 cell death [4, 12, 13]. The success of any HAART combination to cause CD4 T cell repopulation is premised on choosing a regimen that is efficacious, tolerable, and safe in PLWHA [3, 14]. The rate of CD4 T cell repopulation is believed to vary with different HAART combinations and is also affected by both human (age, gender, baseline and CD4 and CD8 count) and viral (baseline viral load) factors [3, 10, 11, 15–17]. The world health organization (WHO) guidelines and Ghana in its 2014 ART guidelines have recommended tenofovir, lamivudine/emtricitabine, and efavirenz combination as the most preferred first line for management of HIV 1 over the most available zidovudine-based and other first-line combinations [18–20]. Both Spaulding et al. and Gallant et al. suggest a superior immune reconstitution and adverse drug reactions/events (ADREs) profile with tenofovir-based regimen relative to zidovudine regimen based on studies conducted in developed countries [3, 12, 21]. However, Joshi et al. and other related studies have shown there exist inter- and intrapopulation difference in response to HAART along with other host and viral factors [3, 6, 10, 14, 15, 21–23]. Since the introduction of tenofovir-based regimen in Ghana in 2014, there has been little or no scientific data that reflect the local scientific reality as a developing country and must be addressed and put into perspective.

This study therefore sought to evaluate and compare the efficacy (immune reconstitution propensity) and safety (adverse drug reactions/events, ADREs) of tenofovir, lamivudine, and efavirenz regimen (tenofovir cohort) to zidovudine, lamivudine, and efavirenz regimen (zidovudine cohort) and further assess how human related factors affect efficacy and safety of these HAART regimens in PLWHA.

## 2. Study Design and Targeted Population

This was a prospective observational study involving only participants who enrolled on a naïve tenofovir, lamivudine, and efavirenz regimen known as tenofovir cohort and those on zidovudine, lamivudine, and efavirenz also known as zidovudine cohort at Komfo Anokye Teaching Hospital (KATH) in 2016. The above-mentioned HAART regimens are the commonly used and readily available first-line therapeutic options for the management of HIV 1 infections at KATH and in Ghana, with tenofovir regimen believed to have a favorable dosing schedule and more tolerable effects than the zidovudine regimen. The only difference between the two cohorts was either the use of tenofovir or zidovudine in the respective cohorts, with all other variables being constant. Also the sampling technique used for selection of patients for participation in the study was systematic random sampling and thus did not interfere with the choice of treatment. The selection of HAART regimen was thus the sole responsibility of the clinician in charge of the adult HIV clinic at the study site, guided by patients' comorbidities, tolerability, and acceptance. A total of 106 patients were recruited for the study estimated from the annual HIV/AIDS cases at KATH. A total of 80 participants were in the tenofovir cohort while 26 were in zidovudine cohort.

### 2.1. Inclusion and Exclusion Criteria

Included in the study were adults aged 18 years and above and newly diagnosed of HIV 1 in 2016. In addition, only those with no past exposure to HAART known as HAART naïve were considered for the study. Furthermore and more importantly, to be considered for the study, one must have been on any two of the commonly prescribed regimens, namely, tenofovir with lamivudine and efavirenz (tenofovir cohort) or zidovudine with lamivudine and efavirenz. Only those with baseline CD4 T cells with or without viral load determined before the initiation of a naive HAART were considered. Those with HIV 2 infection or both HIV1 and HIV 2 were excluded from the study. These were necessary to limit confounding factors and provide similar conditions for a plausible comparison.

### 2.2. Data Collection

Each patient who consented for inclusion in the study was given a unique identification number and allocated a designated location in the data collection notebook in an ordered fashion. Data on demographics and vital signs (age, sex, disclosure, weight, height, lifestyle, herbal medicine use, and blood pressure) were obtained from patients' folders and laboratory reports. Patients were made to undertake CD4 T cell absolute count, viral load, and full blood count before the initiation of naïve HAART. Patients were followed up to assess the occurrence of adverse drug reactions/events especially during the first five weeks after HAART initiation. Study subjects were made to repeat CD4 T cells absolute count using flow cytometer (BD FACS caliber) per manufacturer's protocol (normal reference range 400–1400 cells/*μ*L) at a median period of five months (range 4–6 months) after HAART initiation at the serology unit of the Komfo Anokye Teaching Hospital.

### 2.3. Statistical Analysis

STATA version 12 was used for data analysis. Normality test was carried out on all continuous variables and presented as either mean ± standard deviation or median with range values for normally and nonnormally distributed data, respectively. The within-group variations of the paired differences between CD4 T cell count before and after five months of treatment were compared using Wilcoxon Signed Ranks Test in each cohort and overall effect. The Mann–Whitney *U* test was used to test for differences in CD4 T cell absolute count change across the treatments cohorts and human factors. A multivariate regression analysis was performed to assess predictors of CD4 T cell change after HAART. All categorical variables are presented as frequencies and/or proportions.

### 2.4. Ethical Considerations

Approval was sought from the management of the Komfo Anokye Teaching Hospital. This was to ensure that the hospital has prior knowledge of the research and to affirm if the facility is equipped enough to enable the proper execution of the research work. Ethical Clearance with reference number CHRPE/AP/499/16 was granted by the Committee on Human Research, Publications and Ethics of the Kwame Nkrumah University of Science and Technology, KNUST, and Komfo Anokye Teaching Hospital (KATH) to ensure the research protocol does not breach research ethics involving human subjects. Finally, during recruitment of study subjects, a written informed consent was obtained from the study participants freely, after the purpose of the study has been explained to them, and they or their immediate relations have been assured of client confidentiality and anonymity of data.

## 3. Results

### 3.1. Demographics and Comorbidities

As shown in Table 1, a total of 106 HAART naive HIV patients were enrolled with 89 successfully followed up out of which 68 (76.4%) were in the tenofovir cohort while 21 (23.6%) were in the zidovudine cohort. In this study, there were 65.1% (69) females and 34.9% (37) males. The participants were found to have a mean age of 42.6 ± 11.4. However, females were relatively younger with a mean age of 39.1 ± 11.6 compared to males with mean age of 42 ± 11 with both sexes showing considerable age variability. HIV is known to exist with several comorbidities and, in this study, 34 (32%) had comorbidities. The most prevalent comorbidity was tuberculosis with 21 (19.8%) subjects contracting the disease and this was followed by hepatitis B virus infection, where 7 participants were seropositive to HbsAg. Also in this study, and in accordance with WHO clinical staging of HIV/AIDS at baseline, about 57% of participants had progressed to stage III (CD4 count < 200 cells/*μ*L) with 33% at stage II (200–499 cells/mL).

### 3.2. Immune Reconstitution across Treatment Groups

Out of 106 participants recruited into the study, 17 were lost to follow-up while 89 were successfully followed up for a median period of five months (20th week). From Table 2, after 5 months of initiating a naïve HAART, there was a significantly higher median CD4 T cells count in all subjects 327 (45–972) compared to 142.5 (0–556) cells/*μ*L at baseline (*p* < 0.0001). Also, within each treatment group, there was significant increase in posttreatment CD4 T cells count from baseline after 5 months of treatment. The extent of CD4 count change between baseline and posttreatment was assessed between the two cohorts. Tenofovir regimen (TLE) recorded a significantly (*p* < 0.0001) higher median CD4 cell increase of 214 (0–795) cells/*μ*L compared to zidovudine regimen (ZLE) which recorded a median increase of 109.5 (21–275) cells/*μ*L. Furthermore, the univariate Mann–Whitney tests to determine the CD4 count change between those with and without individual comorbidities and young and older adults were unexpectedly found not to be significantly associated with any difference in CD4 count change after HAART as observed. Interestingly, females were found to have higher median CD4 T cell change 217 (7–516) cells/*μ*L compared to males 149.5 (21–401) cells/*μ*L. However in a multivariate analysis gender and likewise other variables were found not to be associated with CD4 T cell change. Thus the significant change in the CD4 T count recorded among the study participants prior to TLE/ZLE administration within 5 months was independent of age, gender, comorbidities (TB and hepatitis B infections), and baseline CD4 and CD8 cell counts. Contrary to that, the observed CD4+ count change among the study participants varied significantly across the treatment groups.

### 3.3. Safety Assessment of HAART (TLE/ZLE) on Study Participants

**Figure 1:**
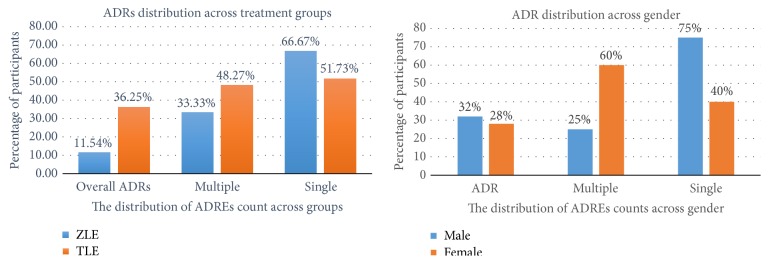
The figure shows the distribution of adverse drug reactions/events (ADREs) across comparison groups. The figure shows how treatment type and gender affect the occurrence of adverse drug reactions/events (ADREs) among participants. ADR refers to the proportion of participants who experienced adverse drug reactions/events in each comparison group. Single: the proportion of persons who had only one ADRE out of the number of persons with ADREs in each group. Multiple: the proportion of persons who had two or more ADREs out of the number of persons with ADREs in each group.

In this study during follow-up, one participant each on TLE and ZLE died with the one from ZLE cohort said to have had Kaposi sarcoma. Two participants on TLE change from efavirenz to nevirapine due to intolerable CNS effects (psychosis) while one patient on ZLE was switched to TLE due to unfavorable dosing schedule and side effects. In terms of serious ADRE, one participant with Kaposi sarcoma in tenofovir cohort experienced tenofovir induced nephropathy and two experience intolerable psychosis.  Figure 1 shows the distribution of adverse drug reaction/events (ADREs) occurrences within the ZLE and TLE treatments groups. In all, 11.4% participants on ZLE treatment experienced ADR compared to 36.3% in the TLE treatment group. Moreover, out of the proportion of participants who reported side effects in each treatment arm, 33.3% of the ZLE treatment group compared to 48.3 percent of the TLE treatment group recorded multiple ADREs.  Figure 1 shows 32% males and 28% females experienced ADREs. However females are more likely to experience multiple ADREs as 60% of females who experienced ADREs compared to 25% of males who experienced ADREs had multiple events.

From Table 3, the most prevalent ADREs category was CNS effects with diverse manifestations accounting for about 33% (dizziness accounted for 12% while other CNS ADREs like nightmare, insomnia, psychosis, and excessive sleep accounted for about 21% of all reported cases). The most prevalent reported single ADRE was skin rash as 20% of reported ADREs were rash with overall cutaneous ADREs accounting for 31.1% of all ADREs. GIT effects were all attributable to tenofovir therapy with about 77% experienced by females.

## 4. Discussion

Many studies support the assertion that HAART enhances immune recovery which partly is reflected as an increase in absolute CD4 T cell count [3, 11]. However this property is believed to be affected by the type of HAART combination and other factors [3, 10, 14, 21]. In this study, participants were initiated on a naïve HAART comprised of lamivudine and efavirenz in combination with either tenofovir or zidovudine. Their propensity to repopulate CD4 T cell and ADREs experienced in PLWHA were subsequently assessed. Close to 56% of the study participants were in stage III which suggests late diagnosis of HIV in these participants. Overall, participants exhibited a significant (*p* < 0.0001) increase in median CD4 T cell absolute count after five months of being on HAART compared to baseline CD4 T cell absolute count before HAART initiation. This study finding agrees with many studies that HAART enhances immune recovery [3, 10, 15, 19].

The extent of CD4 cells change five months to HAART between the two cohorts when assessed. Tenofovir treatment arm yielded a significantly (*p* < 0.0001) higher median increase in CD4 T cell absolute count of 214 (0–795) cells/*μ*l compared to zidovudine treatment arm of 109.5 (21–275) cells/*μ*l five months after HAART. The finding is consistent with a systematic review by Gallant et al. which purported tenofovir-based HAART to elicit superior immune recovery rate than zidovudine-based HAART but an equipotent in virologic suppression [12, 21, 24, 25] while another study found tenofovir to be noninferior to zidovudine in both immune recovery and virologic suppression [12, 21]. From other studies, the ability of any HAART regimen to effect CD4 cell repopulation is premised on enhancing thymic functionality for improve thymopoiesis [3, 15, 25]. Additionally, their ability to reverse HIV-induced chronic immune activation enhancing positive selection of naïve CD4 T cells in the thymus gland is widely acknowledged [4]. Immune reconstitution as a property of HAART has also been associated with the reduction in the cytotoxic effect of* naf*,* vpr,* and* tat* viral proteins on CD4 T cells through the activation of NF-kappa *β* and TNF-*α* [2]. The observed immune recovery in this study may be due to reduction in chronic immune activation, cytolysis, cytotoxic viral proteins, and an increase in thymic function though there is not much data on these parameters for analysis to support this claim [2, 7, 15, 26]. However the observed higher CD4 T cells absolute count increase in the tenofovir cohort compared to zidovudine cohort may be attributable to the postulated zidovudine induced stasis of CD4 T cells expansion. Zidovudine seems to enhance cytotoxic lymphocytes activity leading to infected CD4 T cells death via cytolysis. It also impedes antigen and lectin-induced T cells proliferation and mitosis causing an impaired T cells expansion [17].

The significant change in the CD4+ count recorded among the study participants prior to TLE/ZLE administration was found to be independent of age and HIV comorbidities (TB and hepatitis B infections). In a univariate analysis, gender was associated with CD4 count change as females recorded a significantly (*p* = 0.0094) elevated median CD4 count 217 (7–516) compared to males 149.7 (21–401) cells/*μ*L. However in a multivariate analysis, the association was not statistically significant. This was unexpected as it finds no association between any of the variables evaluated. With regard to age, children and younger adults generally have lager thymic volume reserve at baseline than the elderly and are expected to have superior immune recovery [15, 27]. However this study excluded children and adolescence but only considered young and old adults. Also, more than half of the study participants were in stage III and are normally known to have reduced baseline thymic reserve due to long standing HIV infection [15]. These could account for the fact that there is no significant difference in immune reconstitution between the older and young adults and even across sex.

Despite the enormous gains since the advent of HAART, adverse drug reactions/events continue to be a major setback to the success of most HAART pharmacotherapies. In most instances, noncompliance and treatment discontinuation are due largely to the development of HAART related ADREs in PLWHA [28–33]. An Indian study estimated over 90% PLWHA starting a naïve HAART reacted adversely while a Cameroon-based study reported about 20% of such ADRs [32, 34]. The Lartey et al. study conducted in Ghana revealed ADR prevalence of 9.4% among HAART users with diarrhea and anemia being the commonest [30].

Altogether, ADREs were reported in about a third of the study participants. Those in the tenofovir cohort somehow appeared to have experienced more of the ADRs and multiple adverse events compared to the zidovudine cohort. The unexpected higher incidence of ADREs associated with tenofovir compared to zidovudine can be explained as follows. First, there was high incidence of cutaneous ADREs manifesting as skin rash, itching, and others of varying degrees and this may be attributed to synergistic cutaneous adverse effects of tenofovir and efavirenz as more than two-thirds of the cutaneous ADREs experienced in this study (78%) were in the tenofovir group. Likewise, the most common ADREs category was CNS side effects with over 93% CNS effects experienced by those in the tenofovir cohort. This also reveals a possible potentiated interaction of tenofovir and efavirenz resulting in synergized CNS adverse effects of the tenofovir regimen. Efavirenz and its metabolite 8-hydroxyefavirenz are known to reach higher concentrations in the CSF and likewise tenofovir increasing the risk of neurotoxicity and disturbance of brain bioenergetics from an inconclusive brain mitochondrial polymerase-*γ* activity inhibition and activation of inducible nitric oxide synthetase [15, 28–30, 32, 35]. Only one patient had tenofovir induced nephropathy (TIN) as serious ADR. This TIN is due to the selective mitochondrial toxicity of tenofovir on the renal tubules due to inhibition of MtDNA polymerase-*γ* resulting in acute tubular necrosis [31]. One patient in the tenofovir also reported serious psychiatric disorders resulting in subsequent change to nevirapine. Gender related difference in side effects exists with females estimated to possess higher likelihood of experiencing adverse events to HAART though some found no or contradicting results [14, 23]. There was no significant difference between males and females in this study; however, females appeared to experience more multiple ADRs than males. In all, the unexpected higher ADREs associated with tenofovir compared to zidovudine may be due population variation and synergistic ADREs (cutaneous and neuropsychiatric effects) associated with tenofovir and efavirenz. Also the higher number of participants in the tenofovir cohort and female gender compared to the zidovudine and male gender, respectively, may have also influenced the proportions of ADREs as observed [15, 28–30, 32, 35].

Despite the evidence generated in this study, data on the HIV RNA load, a primary biomarker, could not be obtained for all of the study patients during the follow-up phase in the study, due to breakdown of Cobas Ampliprep HIV 1 analyzer at the facility. If this were done the evidence generated in this study could have been more robust. This study could not also obtain follow-up data for LFT, FBC, and RFT on about two-thirds of the study participants due to financial constraints and hence results on these parameters could not be assessed for more definitive evaluation of safety of the HAART pharmacotherapy. Again the number of participants involved in the study was relatively small (*n* = 106), and this could be attributed to the difficulty the investigators had in getting naïve HAART patients to enroll at the beginning of the study. The individuals who participated in this study however are a reflection of the annual estimate of HAART naïve HIV cases that visited the hospital and the attrition rate is also found to be below the 20% margin [36]. Finally about 17 patients were lost to follow-up and thus could not be traced for assessment. This was believed to be the result of movement of the patients or their relations out of catchment area of the hospital or might have travelled to live in other locations outside the catchment area of the hospital. All these notwithstanding, the evidence generated is interesting and can be described as a true reflection of the safety and efficacy of the various HAART regimen for HIV/AIDs case management and care at the study site.

## 5. Recommendation for Further Research

We therefore recommend a study with a much more robust design such as a multicenter noninferiority trial or a national cohort study to compare the efficacy and safety of tenofovir-based HARRT regimens and zidovudine-based HAART regimens in Ghana. Also we recommend an evaluation of the comparative effect of tenofovir-based and Zidovudine-based HAART regimens on markers of thymic functionality and immune activation in people living with HIV/AIDS.

## 6. Conclusion

The tenofovir-based cohort had a superior immune reconstitution potential compared to zidovudine-based cohort. Patients on the zidovudine regimen also improved significantly with regard to CD4 T cell repopulation. The immune reconstitution potentials of both HAART pharmacotherapy were independent of age, gender, baseline CD4 and CD8 T cells count, herbal medicine use, and HIV related comorbidities. Patients on the tenofovir-based regimen and females reported to have had more ADREs compared to those on zidovudine-based regimen. Although the tenofovir treatment arm in this study showed a superior immunological recovery, its use must be closely monitored to address the occurrence of ADREs to improve compliance.

## Figures and Tables

**Table 1:** Demographic and baseline characteristics of the study participants at diagnosis.

Variable	Observations	Mean age	Std. Dev.	Min	Max
Females	69 (65.1%)	39.09	11.8	20	78
Males	37 (34.9%)	42.30	11.0	22	72

Total	106	42.63	11.4	20	78

Distribution of participants across treatment groups before and during follow-up					

Treatment group	Initial distribution			Follow-up distribution	

TLE (tenofovir cohort)	80 (75.5%)			68 (76.4%)	
ZLE (zidovudine cohort)	26 (24.5%)			21 (23.6%)	

Total	106 (100%)			89 (100%)	

Comorbidities				Number of participants	

Tuberculosis				21	
Hepatitis B				7	
Hepatitis C				1	
Kaposi sarcoma				4	
Cerebral toxoplasmosis				1	

Total comorbidities				34	

Stage of HIV (CD4 count/cells/*µ*L)	Number of participants			Percentage Freq. (%)	

Stage I (≥500)	11			10.4	
Stage II (200–499)	35			33.0	
Stage III (0–199)	60			56.6	

Total number	106			100	

The table shows the baseline characteristics of study participants before and during the initiation of therapy. It shows their age distribution across gender at diagnosis and the comorbidity profile of participants determined before and during treatment with HAART. The stage of HIV was determined at baseline depicting the degree of progression to AIDS using the CD4 T cells count.

**Table 2:** Comparison of CD4 T cells count change between treatment groups and how other variables affect CD4 T cells reconstitution following the initiation of HAART.

Comparison between baseline CD4 count and outcome after median of 5 months after HAART				
Group	Pretreatment cells/*µ*L	Posttreatment cells/*µ*L		*p* value

Overall	142.5 ( 0–556)	327 (45–972)		<**0.0001**
ZLE	104 (0–509)	200 (45–889)		<**0.0001**
TLE	150 (3–556)	337 (69–972)		<**0.0001**

Determination of the effect HIV co-morbidities, gender, and increasing age on with CD4 T cell reconstitution				
Parameter				*p* value

	Gender			
CD4+ count change after 5 months of TLE/ZLE treatment	Male	Female		
	149.7 (21–401)	217 (7–516)		**0.0094**
	Age group (years)			
	20 to 40	>40		
	364.5 (45–972)	352.1 (76–697)		0.3184
	TB status			
	No	Yes		
	190.1 (0–500)	211.9 (21–578)		0.5717
	Hepatitis B status			
	No	Yes		
	172 (0–578)	215.6 (52–319)		0.3059
	Treatment group			
	ZLE	TLE		
	109.5 (21–275)	214 (0–795)		**<0.0001**

Multivariate model to predict the influencing variables on CD4 change after HAART				
*F* (8, 78) = 1.03, Prob > *F* = 0.4176, *R*^2^ = 0.0959, adjusted *R*^2^ = 0.0032				
CD4 change	Coefficient	Standard error	*t*	*p* value

Gender	−89.999	47.207	−1.91	0.060
Age	−0.313	1.758	−0.18	0.859
Hepatitis B	73.777	87.532	0.84	0.402
Tuberculosis	61.796	54.536	1.13	0.261
Baseline CD4	0.066	0.1292	0.52	0.607
Baseline Hb	15.814	11.682	1.35	0.180
Baseline CD8	−0.0123	0.015	−0.82	0.412
Herbs use	−46.145	42.435	−1.09	0.280

From the table, data is presented as median with range values. TLE: tenofovir, lamivudine, and efavirenz and ZLE: zidovudine, lamivudine, and efavirenz. *p* is significant at 0.05. Mann–Whitney test was used to compare the median CD4 cells count increase between the tenofovir treatment group (TLE) and the zidovudine treatment group (ZLE) and likewise the effect age, gender, and comorbidities at univariate level. The last part of the table is a multivariate regression analysis to identify variables that may predict CD4 T cells count change. *t* is the test-statistic, and *p* > *t* is the probability of predictive value of each variable on CD4 T cells count significant at 0.05. *R*^2^ measures the degree of correlation between variables with CD4 T cells count change.

**Table 3:** Classification/distribution of ADREs in the treatment groups and gender.

ADREs description	Overall	Treatment groups		Gender	
		TLE	ZLE	Male	Female
Cutaneous	14 (31.1%)	11 (78.6%)	3 (21.4%)	4 (28.6%)	10 (71.4%)
GIT effects	10 (22.2%)	10 (100%)	0 (0%)	3 (21.4%)	7 (78.6%)
CNS effects	15 (33.3%)	14 (93.3%)	1 (6.7%)	5 (33.3%)	10 (66.7%)
Mitochondrial toxicity	6 (13.3%)	6 (100%)	0 (0%)	1 (16.7%)	5 (83.3%)

Total	45 (100%)				

^*∗*^Serious ADREs observed	3	3	0	0	3

The table shows the distribution of the specific ADREs experienced at each level of comparison under four broad classification.
